# A7DB: a relational database for mutational, physiological and pharmacological data related to the α7 nicotinic acetylcholine receptor

**DOI:** 10.1186/1471-2202-6-2

**Published:** 2005-01-20

**Authors:** Steven D Buckingham, Luanda Pym, Andrew K Jones, Laurence Brown, Mark SP Sansom, David B Sattelle, Philip C Biggin

**Affiliations:** 1 Laboratory of Molecular Biophysics, Department of Biochemistry, University of Oxford, South Parks Road, Oxford OX1 3QU. UK; 2 MRC Functional Genetics Unit, Department of Human Anatomy and Genetics, University of Oxford, South Parks Road, Oxford, OX1 3QX. UK

## Abstract

**Background:**

Nicotinic acetylcholine receptors (nAChRs) are pentameric proteins that are important drug targets for a variety of diseases including Alzheimer's, schizophrenia and various forms of epilepsy. One of the most intensively studied nAChR subunits in recent years has been α7. This subunit can form functional homomeric pentamers (α7)_5_, which can make interpretation of physiological and structural data much simpler. The growing amount of structural, pharmacological and physiological data for these receptors indicates the need for a dedicated and accurate database to provide a means to access this information in a coherent manner.

**Description:**

A7DB  is a new relational database of manually curated experimental physiological data associated with the α7 nAChR. It aims to store as much of the pharmacology, physiology and structural data pertaining to the α7 nAChR. The data is accessed via web interface that allows a user to search the data in multiple ways: 1) a simple text query 2) an incremental query builder 3) an interactive query builder and 4) a file-based uploadable query. It currently holds more than 460 separately reported experiments on over 85 mutations.

**Conclusions:**

A7DB will be a useful tool to molecular biologists and bioinformaticians not only working on the α7 receptor family of proteins but also in the more general context of nicotinic receptor modelling. Furthermore it sets a precedent for expansion with the inclusion of all nicotinic receptor families and eventually all cys-loop receptor families.

## Background

Nicotinic acetylcholine receptors (nAChRs) are the most studied members of the cys-loop family of ligand-gated ion channels (LGICs) which also contains γ-aminobutyric acid (GABA) receptors, glycine receptors and 5-HT_3 _receptors [1]. Distinct subtypes of nAChRs mediate, for example, fast synaptic transmission in the brain and at neuromuscular junctions [2]. All are believed to be pentameric assemblies of various combinations of different subunits (α, β, γ/ε, δ), some of which exist as multiple isoforms [3]. nAChRs are important targets for novel analgesics as well as new drugs being devised for Alzheimer's disease and schizophrenia [4,5]. Mutations in nAChRs are associated with certain forms of epilepsy [6,7] and several congenital myasthenias [8]. There is a substantial and growing body of physiological, pharmacological, genomic structural and modeling data on receptors formed from these subunits. The volume and diversity of these data present severe challenges for their efficient storage and interpretation. Here we describe a relational database, initially relating to the α7 subunit, whose aim is to provide an easy to use, extensible web-based interface to access functional data and relate it back, wherever possible, to structural data.

## Construction and content

**Figure 1:**
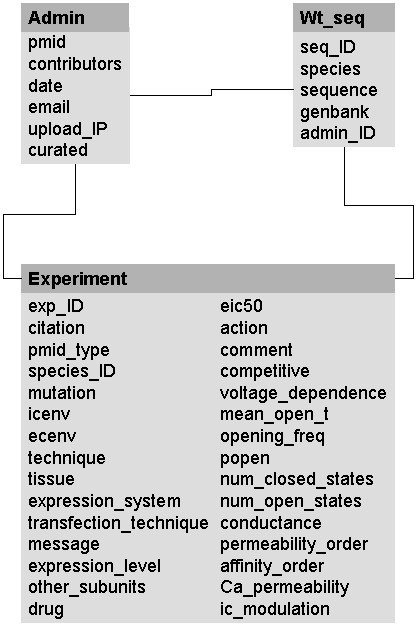
The A7DB database scheme. All the information pertaining to an experiment is stored within a single table called "experiment". The sequence table stores all the wildtype sequences for the different species contained within the database. The third table is the admin table which makes entries and changes traceable. A full description of each of the entries is supplied as supplementary information.

The database is currently limited to nAChR α7 subunits. This makes the initial task of populating the database manageable. To allow 3^rd ^level normalization, the principal information prototype stored in the database is referred to as an "experiment event". An experiment event is a collection of simultaneous measurements and their associated measurement conditions. For example, two drugs tested against the wild type α7 and a mutant under the same experimental conditions would be described by 4 (2 drugs × 2 sequences) experiment events. If the identical measurements were repeated in, say, calcium-free saline, this would yield 8 experiment events. Using this prototype a unique set of experimental conditions is associated with a single set of data. Therefore, the database tables (see Figure 1) have been designed around the central experiment table [see Additional file 1]. Quite often, several real experiments are performed within any one publication. We wanted to capture all of that data. Thus, all the data for one experiment that was reported have been stored (non-redundantly) as a separate entity. The database is managed by the MySQL relational database management system. PHP scripts query the MySQL system and transform the data into HTML pages for serving to the client.

The database currently aims to store molecular information rather than whole-organism genetic information which can be sourced from elsewhere, see for example [9,10]. A typical set of experiments reported in one paper might concern the properties of one mutation at a particular position and its associated changes in physiology and/or pharmacology. All of this data is held in the database.

By use of appropriate alignments (either stored within the server or uploaded) the positions of these data can be highlighted on a homology model based on the acetylcholine binding protein AChBP [11]. The model is viewable by either using the Chime plugin, or by setting the browser to use Rasmol as a helper application. User instructions for configuring different browsers are provided on the server, as well as the list of combinations of operating system and browser that we have tested to date.

For any database, data integrity will ultimately determine its usefulness. The initial population of the database has been carried out by the authors, but it is desirable and practical in the long term to have a procedure whereby any laboratory can submit data. To help ensure reliable deposition procedure, each depositor will have a unique identifier (thus making the data accountable). A depositor will submit their data which is then tabulated and presented as a form which the depositor is then asked to confirm as being correct. Only when this confirmation is received is the data committed to the database. Although this will reduce some entry-based mistakes, the database will nonetheless need to be curated to maintain integrity.

The database does not attempt to duplicate the functionality of other databases. For example we store a local alignment of α7 subunits taken directly from pfam [12], but we do not perform any alignments ourselves. Such tools are already available on numerous web-servers and in any case the user may wish to upload their own alignment. Wherever possible, we link to a well-established resource, as is the practice in for example, SWISS-PROT [13].

## Utility and discussion

Access to the database is through various routes. The first route is by a very simple search string (e.g. citation = Smith*). The user is then presented with a page informing him of the number of hits and asking what information should be displayed from those hits. The second is a simple incremental search in which the user applies criteria sequentially until he chooses to examine the results. As the query is built, the number of hits returned by the query in its current state is shown. The third search method is also incremental but via the use of a set of tabbed pages which divide the information into intuitive categories such as pharmacology and physiology. Additionally, the user can make choices about alignments used and even upload their own. As the query is built, summary information of its status is also displayed. The fourth method to access the data is provided by a 'fast lane' route which provides more experienced users with a quicker and more direct route to the data of interest. The user formulates and constructs a query offline and then uploads it as a simple ASCII file. A sample query form and details of the format are presented in the supplementary information.

The result of a query is presented in tabular form. Only the information requested is actually presented with the option for further or refined searches. In addition to this, the results of any position matches can be displayed via the homologous 3-dimensional structure of the AChBP. This is automatically available if the user selects the alignments available from the server. If the user uploads their own alignment the model will only be produced if AChBP was included. The server streams a Rasmol script or spawns a Chime window depending on browser configuration. In addition to these query routes, the user is also able to browse the contents of the database.

The current database has been designed such that it could easily be extended to include other nAChR subunits and then on to other members of the cys-loop ligand-gated ion channel family. We are currently pursuing this aspect. Such expansion will allow trends that link structure to function in this receptor family to be more readily identified.

The database is very complementary to some existing resources, in particular the LGICdb [14,15]. This database contains sequence, phylogenetic data and sets of coordinates, but does not attempt to store all aspects of individual experiments as we report here. However, this could be used in conjunction with our database to for example explore equivalent positions in related receptors using sequence and phylogenetic information stored therein. Our approach is somewhat similar to that employed by the ProTherm database [16] which aims to store thermodynamic data for mutant and wildtype proteins [17]. Although currently there are no entries common to both databases, it is envisaged that thermodynamic data reported for either the α7 nAChR receptor or related channels will appear in the ProTherm database and can thus be used in conjunction with the A7DB to help illustrate for example how loss of function might be related to protein stability. Furthermore, the similarity in design of these databases should make cross-interrogation possible in the future. We should state that the main difference between A7DB and ProTherm is that instead of thermodynamic data we store pharmacological and physiological data and in that sense it is closer to the voltage-gated potassium channel database [18,19]. Finally, the A7DB is also complementary to the Protein Mutant Database [20] where mutations across many proteins are stored but the searching and data tabulation is primarily text-based. This might be particularly useful if another mutation had been reported in a different protein that bound a similar ligand, for example in acetylcholine also binds to acetylcholinesterase, which we do not store data for.

## Conclusions

We have shown here how the collation and careful storage of experimental data pertaining to one sub-family (the α7 nAChR sub-family) of the ligand-gated ion channels can be assembled into a useful resource. However, the real power of the database will be when it is combined with machine-learning technologies to explore complex relationships between sequence, structure and function [21]. We believe that this resource will grow in usefulness as the amount of data increases. For example, during the construction of this database, atomic coordinates were released for the transmembrane region of the nAChR from *Torpedo marmorata *[22]. Its development is particularly timely given these recent structural developments which allow three-dimensional information to be correlated with function derived from an ever-increasing body of experimental data.

## Availability and requirements

The database is freely available at: 

## Authors' contributions

The project was conceived and technical aspects managed by PCB. The coding and design was done by SB. MSPS and DBS provided critical evaluation of the design and construction process. DBS, LP, AKJ and LB populated the database. All authors have approved the manuscript.

## Supplementary Material

Additional File 1 Supplementary information. This doc file provides a complete description of the table entries and also provides a description of the uploadable query file. Further information about the online help is also provided. Click here for file
