# Supplementary material for: A7DB: a relational database for mutational, physiological and pharmacological data related to the α7 nicotinic acetylcholine receptor
Source: BMC Neurosci. 2005 Jan 20;6:2. doi: 10.1186/1471-2202-6-2 (PMC547909; doi:10.1186/1471-2202-6-2)
Supplement: Additional File 1 — Supplementary information. This doc file provides a complete description of the table entries and also provides a description of the uploadable query file. Further information about the online help is also provided. [file 1471-2202-6-2-S1.doc]

***Supplementary Information.***

There are three tables and the inter-relationships conform to the third normal form (3NF). This means that none of the non-identifying attributes for each entity is duplicated or depends on another non-identifying attribute. There is also an update log that simply records what additions were made to the database. There are three tables:

1. admin.

2. experiment.

3. wt_seq.

Admin table contains the following entries:-

1. pmid: The primary identifier. A unique auto-incrementing integer

2. contributors: The names and addresses of who input the data

3. date: The date, including the time, when they entered the data

4. email: The email address(es) of the person(s) that entered the data

5. upload_IP: The IP address of the computer from which the person(s) uploaded the data. An extra level of accountability so that the inputter can be traced.

6. curated: A Boolean that is set to 0 until the data have been curated, after which it is set to 1. Quality control.

The experiment table is the main data table. The data design is based around the concept of the experiment. A research paper may consist of one or more experiments. An experiment is taken to refer to a set of linked (simultaneous) observations on the phenotype of an expressed wild-type or mutated α7 receptor in a given experimental environment. For example, if the EC50 of ACh was measured for three mutants, this would be represented by three experiments. If the opening frequency was also measured, this would still be three experiments. If, however, the same experiments were repeated in a different bathing medium, there would be six experiments altogether. The experiment table consists of the following columns:

1. exptID: The primary key.

2. citation: A string representing the citation abbreviated to author[etal]year.

3. pmid type: The pubmed identification number. Permits a direct link to pubmed.

4. species_ID: A secondary link to entries in the wt_seq table.

5. mutation: A string representing the mutation. It is of the form [[original amino acid]position substituted amino acid] string where string can be eg. GFP. The retrieval scripts look for the position value when searching for mutations at a given location, and know how to find this in strings of this format. The scripts are also capable of handling multiple mutation entries, such as "A302S, F398Y" provided the entries are separated by commas. Any other entry format would fall through the scripts. Future work should include increasing the capabilities for intelligently searching this column.

6. icenv: The "intracellular environment". Most relevant to patch, represents the composition of the cell interior, ie the patch pipette composition.

7. ecenv: The "extracellular environment". Mainly meaning the bathing medium.

8. technique: [TEVC|SEVC|sharp electrode voltage recording|WC patch|Cell-attached|inside-out|outside-out|Ca imaging|extracellular recording|voltage-sensitive dye]

9. tissue: The tissue used in in-situ recordings, or the origin of the message in heterologous expression.

10. expression_system: The cells used to express in heterologous expression experiments. EG oocytes, S2, HeLa.

11. transfection_technique: The means whereby the message was introduced into the cells in the case of heterologous expression.

12. message: [cRNA|cDNA|mRNA]

13. expression_level: A representation in free form of the level of expression obtained. eg. an experiment might report that a mutation abolished or enhanced expression.

14. other_subunits: Other subunits co-expressed with the α7.

15. drug: The drug (including natural ligands) used in the experiment.

16. eic50: The ec50 or the IC50 as appropriate, expressed in micromolar.

17. action: The action of the compound. [agonist|antagonist|modulator]

18. comment: A bin for text information that may be important in understanding the meaning of the experiment record.

19. competitive: [competitive | non-competitive]. Applies only to antagonists.

20. voltage_dependence: [dependent | independent]. Applies only to antagonists.

21. mean_open_t: The mean open time of the receptor in ms.

22. opening_freq: A free form description of the effect of the mutation+drug on the opening frequency. eg. increased, no change.

23. popen: A free form description of the effect of the drug/mutation on the probability of opening.

24. num_closed_states: The number of closed states.

25. num_open_states: The number of open states.

26. conductance: The effect of the mutation / drug on the conductance of the channel.

27. permeability_order: The order of ionic permeability of the channel.

28. affinity_order: The order of affinity or efficacy of drugs.

29. ca_permeability: The calcium [permeability of the channel in free form.

30. ic_modulation: Freeform description of the modulation of the channel by intracellular factors.

The wt_seq table stores the wild type sequences of the α7 receptors for each species. To allow default amino acid position equivalence across species, the table stores aligned sequences with gaps. These are PFAM alignments. The user has the option of using his own alignments. Scripts reading from this table have the capacity to remove the gaps from the sequences and to take them into account when interpreting amino acid position.

The wt_seq table contains these columns:

1. seq_ID: The primary key identifying the record.

2. species: The species in binomial form.

3. sequence: The amino acid sequence of the α7 wild type.

4. genbank: The genbank entry for this α7.

5. admin_ID: A secondary key linking this to the information on who entered the data.

***The “fast lane” a7db format file.***

A query can be built offline and uploaded in a file that consists of two fields; an identifier and an associated value. A full list of identifiers and their definitions can be found at: http://www.lgics.org/a7db/help/help_search_format.html

An example query file might be:-

mutation_position : 247

citation : revah

expression_system : oocyte

drug : ach

species : Gallus gallus

***Further online information and documentation.***

We have provided a simple run through to demo the typical way one might query the database at :- <http://www.lgics.org/a7db/help/help.html>

There is an accompanying viewlet which demonstrates what you should see at:-

<http://www.lgics.org/a7db/help/a7db_demo_viewlet_swf.html>

A full description of a7db file format to query the database via the “fast lane” is:-

<http://www.lgics.org/a7db/help/help_search_format.php>

Advice on browser configuration is provided at:-

<http://www.lgics.org/a7db/help/browsers.html>

*Related Useful Sites*

LGICdb: The ligand-gated ion channel database can be found at:-

<http://www.ebi.ac.uk/compneur-srv/LGICdb/LGICdb.php>
